# The discovery of the Flammer syndrome: a historical and personal perspective

**DOI:** 10.1007/s13167-017-0090-x

**Published:** 2017-05-22

**Authors:** Josef Flammer, Katarzyna Konieczka

**Affiliations:** Department of Ophthalmology, University of Basel, Mittlere Strasse 91, CH-4031 Basel, Switzerland

**Keywords:** Flammer syndrome, Primary vascular dysregulation, Vasospasm, Glaucoma, Retinitis pigmentosa, Multiple sclerosis, Prediction of health problems

## Abstract

This review describes the clinical and basic research that led to the description of Flammer syndrome. It is narrated from a personal perspective. This research was initiated by the observation of an increased long-term fluctuation of visual fields in a subgroup of glaucoma patients. As these patients had strikingly cold hands, peripheral blood flow was tested with a capillary microscopy, and vasospastic syndrome (VS) was diagnosed. Further studies on these patients revealed frequently weakened autoregulation of ocular blood flow and increased flow resistivity in retroocular vessels. Their retinal vessels were more rigid and irregular and responded less to flickering light. Holistic investigation demonstrated low blood pressure, silent myocardial ischaemia, altered beat-to-beat variation, altered gene expression in the lymphocytes, slightly increased plasma endothelin level and increased systemic oxidative stress. This combination of signs and symptoms was better described by the term primary vascular dysregulation (PVD) than by VS. Subsequent studies showed additional symptoms frequently related to PVD, such as low body mass index, cold extremities combined with slightly increased core temperature, prolonged sleep onset time, reduced feelings of thirst, increased sensitivity to smell and also for certain drugs and increased retinal venous pressure. To better characterise this entire syndrome, the term Flammer syndrome (FS) was introduced. Most subjects with FS were healthy. Nevertheless, FS seemed to increase the risk for certain eye diseases, particularly in younger patients. This included normal-tension glaucoma, anterior ischaemic optic neuropathy, retinal vein occlusions, Susac syndrome and central serous chorioretinopathy. Hereditary diseases, such as Leber’s optic neuropathy or retinitis pigmentosa, were also associated with FS, and FS symptoms and sings occurred more frequent in patients with multiple sclerosis or with acute hearing loss. Further research should lead to a more concise definition of FS, a precise diagnosis and tools for recognizing people at risk for associated diseases. This may ultimately lead to more efficient and more personalised treatment.

## Introduction

Flammer syndrome (FS) [1] describes the phenotype of people with a predisposition for an altered reaction of the blood vessels to stimuli like cold, emotional stress or hypoxia. Whilst FS has clinical implications in many different medical fields, it emerged originally from ophthalmology, particularly from glaucoma research. Over a span of 40 years, we studied, together with our colleagues, the interrelationship of numerous symptoms and signs and their reference to diseases. This finally led to the description of the FS. This review describes the many steps and milestones of the discovery. With growing knowledge, the terminology changed from vasospasm over vasospastic syndrome (VS) to primary vascular dysregulation (PVD) and finally to FS. The syndrome itself has already been comprehensively described [1–3] and is therefore not the principal content here. The focus of the present review is rather on the history of the discovery and particularly on our own personal experience and contribution during this evolution.

## Visual field testing and ocular blood flow

**Fig. 1:**
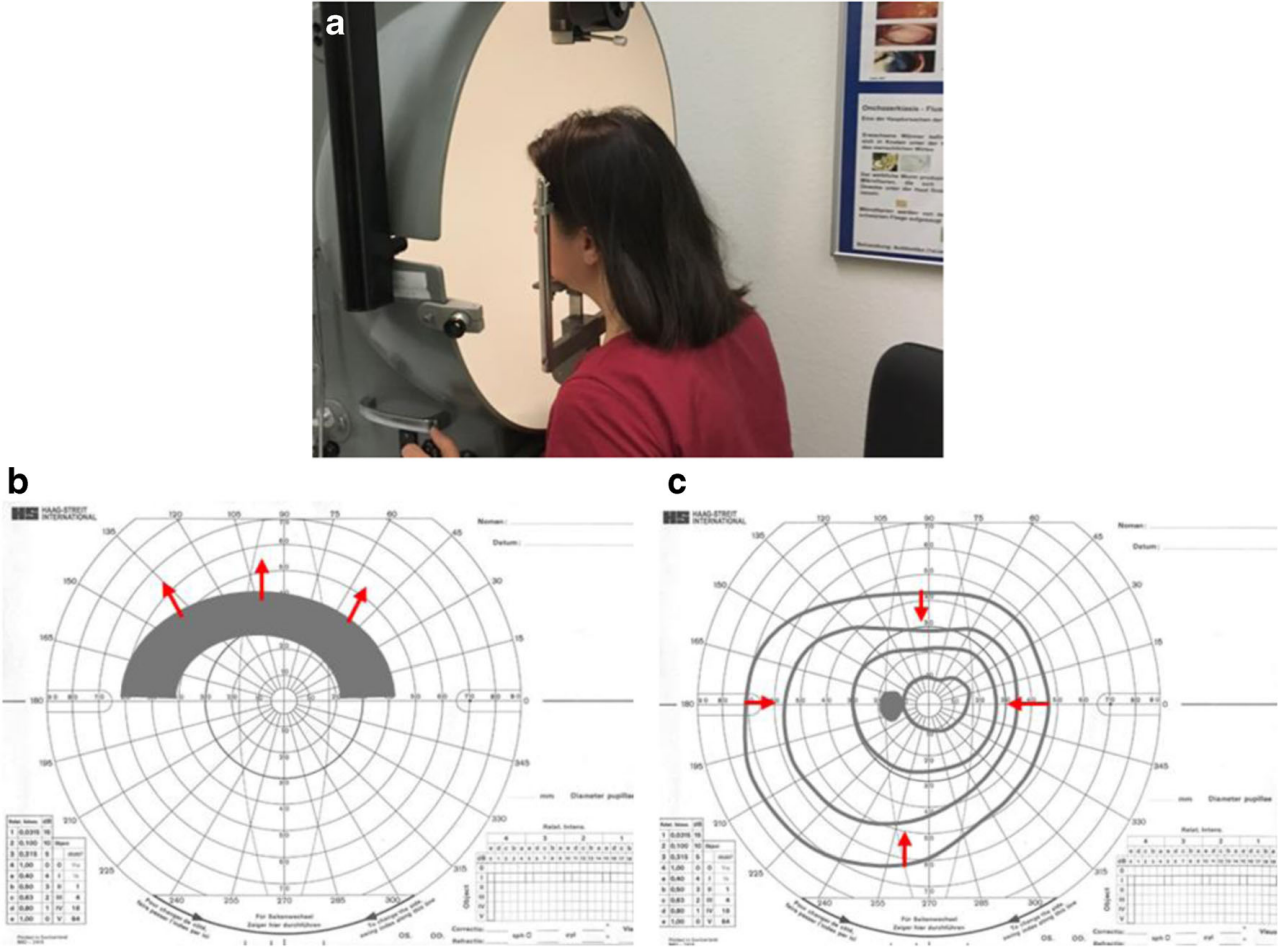
Long-term follow-up of glaucoma patients with Goldmann perimetry (**a**) revealed that both the increase of scotomas (**b**) and the diffuse reduction (**c**) of the differential light sensitivity were highly related to IOP fluctuations

**Fig. 2:**
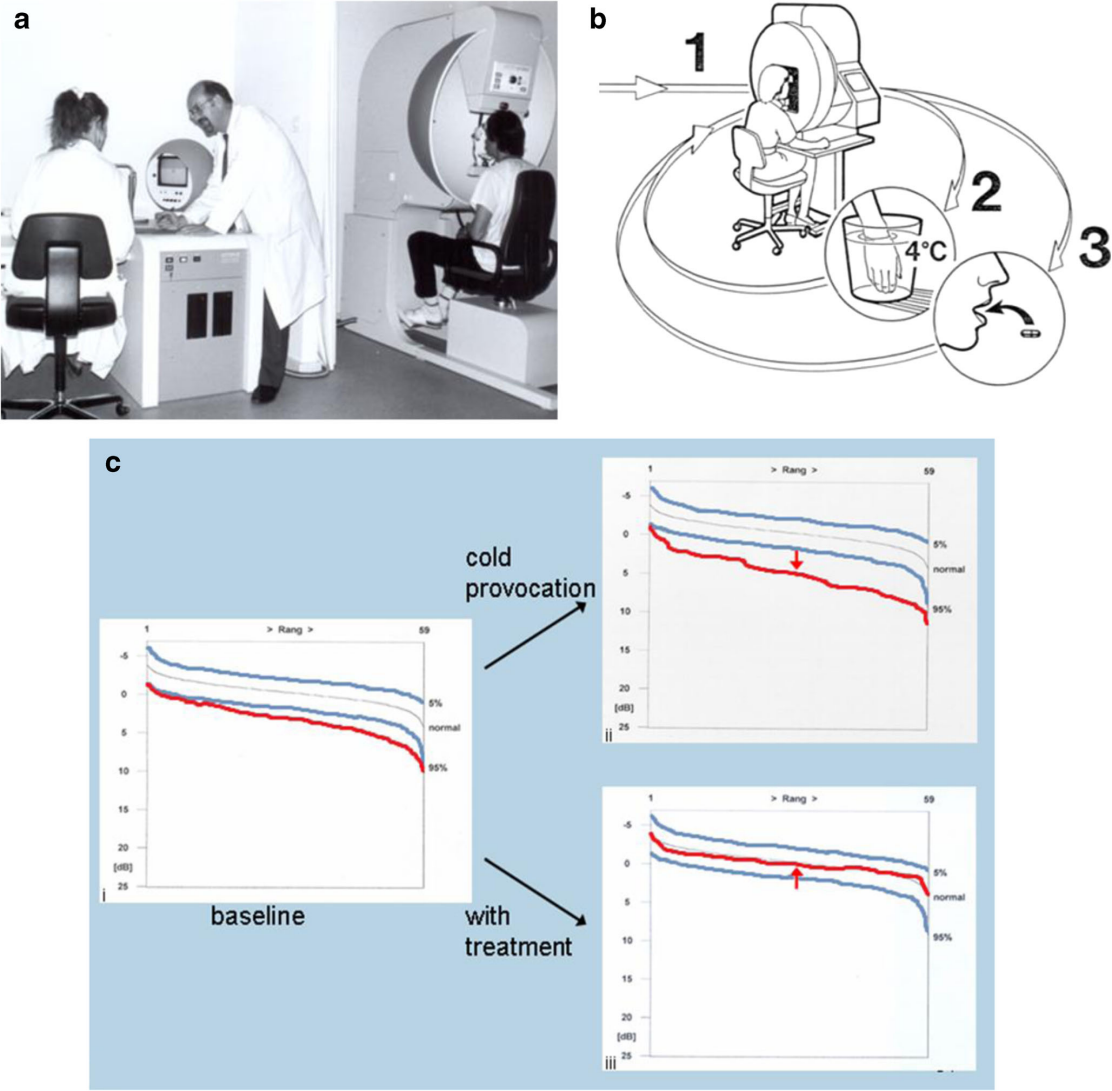
Perimetry. (**a**) Octopus instrument of the first generation. (**b**) The tests were done with the help of the program G1, and the results were presented with the Bebie curve (**c**). Patients with vasospastic syndrome had slight diffuse depressions of the light sensitivity at baseline; these depressions reversibly increased after cold provocation (**c**) and were reversed after treatment with acetazolamide or low dose of a calcium channel blocker (modified after ref. [205])

Interestingly, the very first roots of FS originated from research in perimetry in the early 80s when quantification of ocular blood flow (OBF) was difficult to obtain. To explain this, we must first highlight some steps in the development of perimetry. One of the authors (JF) was trained at the university of Bern, where Hans Goldmann had developed the Goldmann perimetry [4] (Fig. 1a) and Franz Fankhauser had developed the first automated perimeter [5] (Fig. 2a), the Octopus [6]. A study with Goldmann perimetry demonstrated that the fluctuation of intraocular pressure (IOP) from day to day was at least as important for progression of the glaucomatous optic neuropathy (GON) as the average IOP [7, 8] (Fig. 1b,c). We were initially puzzled by this observation and could only explain this finding many years later by OBF instability and oxidative stress [9–11]. After switching to automated perimetry [12], we realised that the visual field defects, particularly the ones from glaucoma, were both more variable and less stable than expected. Bearing this variability in mind, we developed an Octopus program with integrated statistical methods evaluating the visual fields [13]. This research tool served later as a basis for the development of the Octopus program G1 designed for clinical application [14–16]. We established normal values, accounting for age [17] and topography [18], introduced visual field indices to quantify visual field defects [16, 19, 20], compared the index ‘mean defect’ with the ‘mean deviation’ that was introduced later [21] and found some information profit by retests [22], but not by probability weighting [23, 24]. We separated diffuse from local damage with the cumulative defect curve [25], called the Bebie curve [26], and compared it with other methods to quantify diffuse damage [27]. We also studied learning and fatigue effects [28, 29], the correlation between quantitative perimetry and colour vision scores [30], spatial contrast sensitivity [31] and the optic nerve head morphology [32]. After the introduction of the opacity lens meter [33–37], we studied the influence of cataract density on the visual fields [38–40]. We observed visual field reversibility, e.g. improvement in visual performance after radiation of optic neuropathy that resulted from Graves’ disease [41]. We also tested a semi-automated pupillometry on the Octopus [42] and analysed the sources of error in automated static perimetry [43, 44] and the diagnostic role of the peripheral visual field [45]. Our main interest focussed, however, on the short- and long-term fluctuations of the visual fields [46–49]. We realised that some patients had extensive large long-term fluctuation of the diffuse but much less of the local component of the defects [50, 51], as it can particularly be observed with the help of the Bebie curve. We were again confronted with a finding we could not explain. Although this fluctuation was, to some extent, related to the reaction time of the patients [52], it could not be explained by changes in the vigilance of the patient, as neither diazepam [53] nor alcohol [54] had any influence. However, cooling of the hands reduced [55] and treatment with acetazolamide improved visual sensitivity [56] in a subgroup of patients (responders) (Fig. 2b,c). These changes occurred unrelated to IOP [57]. This led to the hypothesis that the long-term fluctuation of visual function could be, at least partly, a consequence of fluctuating OBF [58–61], a concept which was harshly attacked by established scientists in the field. This hypothesis was later supported by the observation that vasodilation by breathing CO_2_ [62] or treatment with calcium channel blockers (CCBs) [55, 63] also improved the visual fields in these types of patients. In other words, the patients with large long-term fluctuation were also the responders to cold and to acetazolamide and CCBs. But why did some patients respond and others did not? [64]. What were the characteristics of the responders? A more detailed consideration of individual cases provided some clues.

## Glaucoma patients with cold hands

**Fig. 3:**
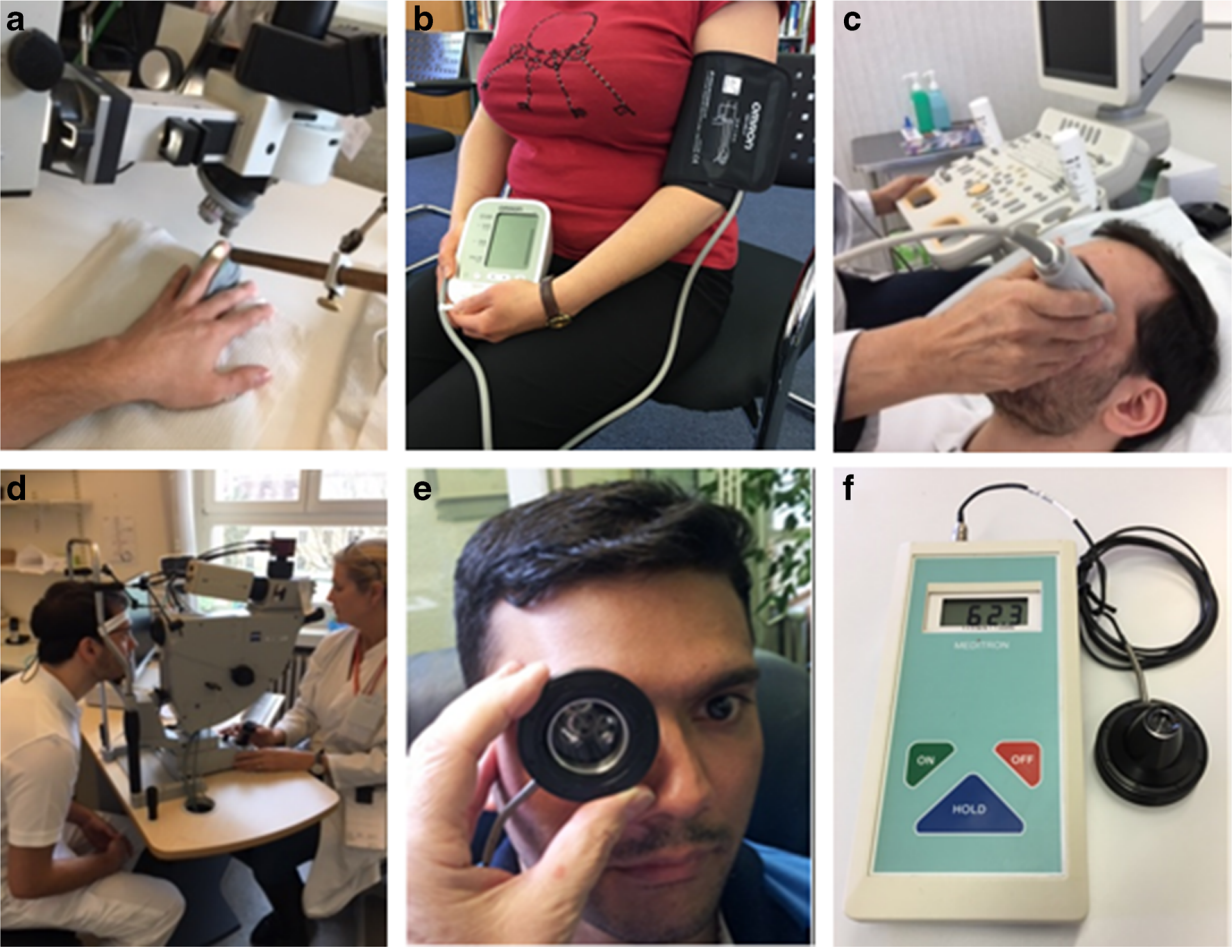
Quantification of aspects of ocular blood flow. With time, new methods were introduced step by step and contributed to the understanding of the Flammer syndrome. Capillary microscopy with cold provocation (**a**), 24-h blood pressure monitoring (**b**), colour Doppler imaging (CDI) of the retroocular vessels (**c**), static and dynamic analyses of the diameter of retinal arteries and veins at baseline and after flicker light stimulation (**d**) and quantification of retinal venous pressure (**e**, **f**)

In the early 80s, a 45-year-old, slim, intellectual and sportive lady with progressive normal-tension glaucoma (NTG) came to see JF for a second opinion. She reported that her internist attested her to be of excellent health. However, her hands were strikingly cold, and, keeping the perimetry studies in mind, we asked whether this may somehow be related to her NTG. Although she emphasised that this was totally normal for her and that her mother also had cold hands, she agreed to be tested by nailfold capillaroscopy. The cold provocation led to a prolonged flow cessation in the nailfold capillaries and the angiologist diagnosed VS [65] (Fig. 3a).

### Glaucoma patients with VS

We knew now that this patient had two conditions: VS and an NTG. What we did not know was whether these two conditions were related. When treating the VS with a CCB, not only did her hands became notably warmer but the visual fields also improved. After observing additional similar cases, we postulated that VS could be a risk factor for NTG [66]. At that time, our hypothesis was considered absurd by most experts, but the findings impressed Dr. S.M. Drance, the former teacher of JF. He visited our lab and subsequently confirmed our observation using another method, namely a laser Doppler flowmeter to quantify BF in the finger [67]. Our own studies with capillaroscopy reconfirmed the longer flow cessation after cooling and reduced blood flow velocity [68] in a subgroup of NTG patients. The induced visual field changes correlated with the blood flow changes in the finger [69], reaffirming that the outcome of perimetry may depend on OBF. Years later, we were able to confirm a relationship between OBF and finger blood flow [70]. However, this correlation existed only in the subgroup of patients with VS, not in subjects without VS.

Taken together, subjects with VS responded to provocation and treatment with both finger blood flow and the visual fields. These responders also seemed to be at greater risk for NTG and potentially for other eye diseases [58, 71]. But one problem at that time was this: we could only postulate the involvement of ocular blood flow (OBF) in VS, and we were not able to measure it directly [72]. Our theory therefore remained vulnerable.

### Systemic hypotension, a component of the VS

Whilst we know today that arterial hypertension increases the risk for ocular hypertension and thereby also the probability for high-tension glaucoma (HTG), arterial hypotension increases the risk for GON at a given IOP. The question of the role of systemic blood pressure (BP) in glaucoma was a matter of hot debate at that time. Some authors claimed hypertension and others hypotension to be related to glaucoma, yet others claimed that BP played no role at all. We monitored 24-h blood pressure [73] and found many glaucoma patients with low BP, particularly at night (Fig. 3b). In fact, it turned out that both HTG patients with progression despite well-controlled IOP and NTG patients had significantly lower BP [74]. The lower the BP was, the longer the flow cessation in the nailfold capillaries after cold provocation was [75]. The endothelin sensitivity correlated positively with the apolipoprotein B concentrations [76] but inversely with BP [77]. Although VS and arterial hypotension independently increased the risk for GON [78], the prevalence of these two risk factors was clearly related. In other words, arterial hypotension was a frequent sign of VS. If BP was too low, we asked patients to increase their salt intake and in extreme cases treated them with fludrocortisone [79, 80].

### Increased blood flow resistivity, an additional component of VS

In the early 1990s, colour Doppler imaging (CDI) reached a resolution that made it possible to measure blood flow or at least flow velocity in retroocular vessels (Fig. 3c). We established normal values [81], frequency distribution [82] and reproducibility [83]. We then observed an increased flow resistivity, not related to IOP, in a subgroup of glaucoma patients [84]. Although this increase in resistivity was not specific for VS [85], it could not be explained by hypercholesterolemia [86] or smoking [87], indicating that arteriosclerosis was not likely the cause. However, the resistivity was inversely related to BP [75, 88] and patients with nocturnal dips [88] and with VS [89] had higher resistivity. Taken together, this was a strong indication that VS also affected the ocular blood flow and that a number of drugs, such as dipyridamole [90], could reduce this flow resistivity.

## Regulation of ocular vessels in ex vivo studies

**Fig. 4:**
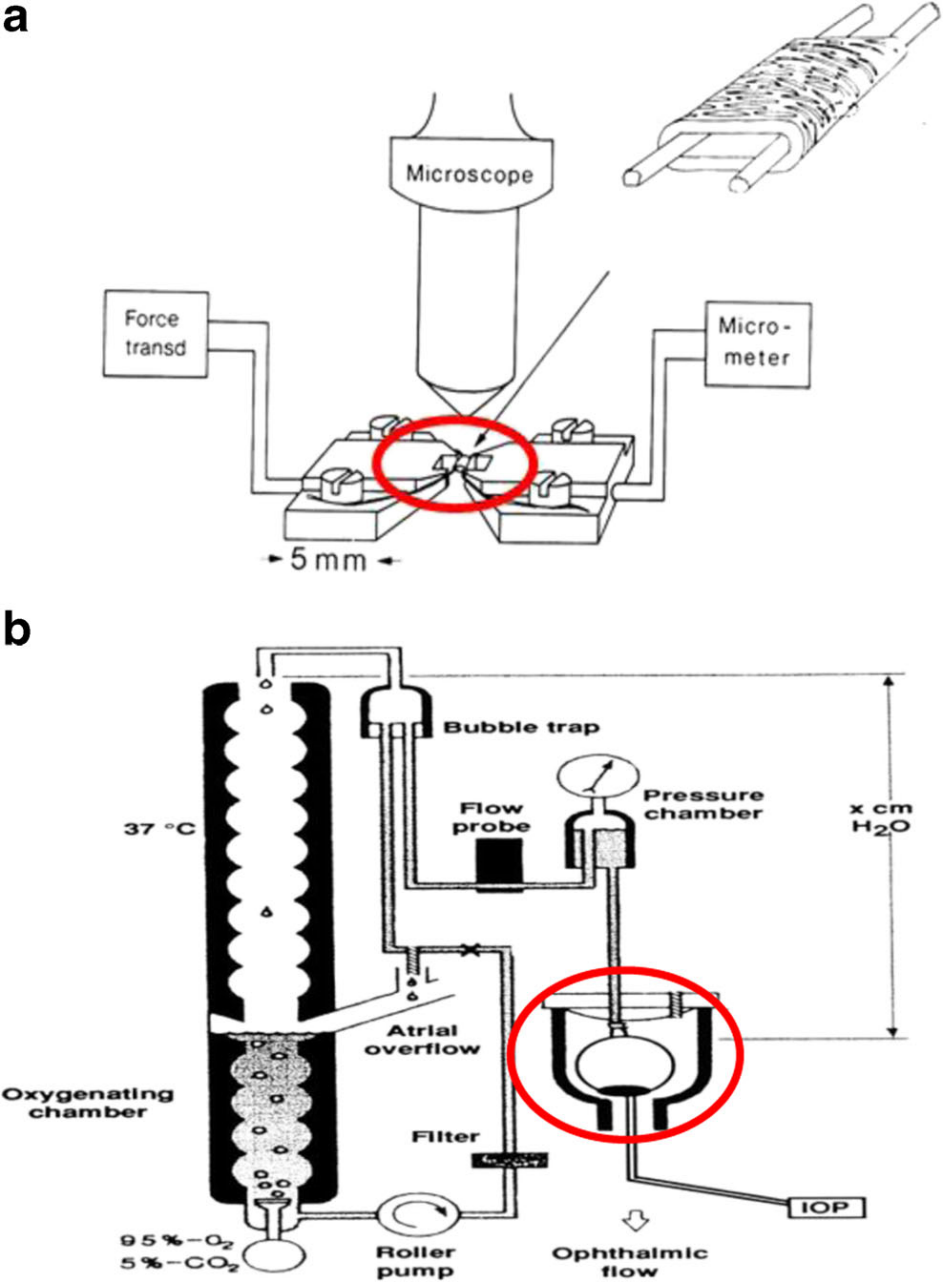
Physiological and pharmacological ex vivo testing of ocular vessels, with the help of a myograph system (**a**) and the help of a perfused eye model (**b**) (modified after ref. [141])

We also studied the physiology and pharmacology of the eye [91–113]. The involvement of OBF in VS motivated us to study the regulation of OBF in particular. We isolated retroocular vessels and studied them with the myograph system (Fig. 4a). Using this experimental setup, we described the endothelium-dependent regulation of vascular tone [114, 115], including its heterogeneity [116], the role of nitric oxide and endothelin (ET) [117] and the refractoriness to the effect of ET [118]. We described the localisation of AT1- [119, 120], melatonin- [121] and melanin-concentrating hormone receptors [91] and characterised the influence of local anaesthetics [122]. Further studies explored the effect of ACE inhibitors and angiotensin receptor antagonists [123], of CCBs [124] at high and low doses [125], dipyridamole [126], potassium-channel blockers [127] and openers [128], magnesium [129], ox-LDL and the ET antagonists BQ 123 [130] and avosentan [131], bimatoprost [132], carteolol [133, 134], PGF2alpha [135] travoprost [136], latanoprost [137, 138], melatonin [139] and adrenomedullin [140]. We also studied the regulation of the vortex vein [101]. Findings with isolated vessels were then also confirmed in a perfused eye model [141] (Fig. 4b). Other groups studied the retinal arteries [142] and veins [143].

Taken together, we learned (i) that ocular vessels were highly regulated, particularly by the vascular endothelium cells, and therefore, a dysregulation due to an endotheliopathy was conceivable; (ii) that the regulation was different from one vessel to another and there was even inhomogeneity within the same vessel; and (iii) that many different drugs influenced OBF. On the one hand, this opened the door for new therapeutic strategies, but on the other hand, it made it necessary to ask patients for local and systemic drugs they were using when interpreting OBF [144–150].

## BF is influenced by local and systemic factors

The optic nerve head (ONH) was of special interest in diseases like glaucoma. However, measuring blood flow (BF) in the ONH was difficult [151, 152]. It was therefore of interest to know whether BF in different tissues was related. Indeed, ONH BF correlated with retrobulbar [153] and finger BF [70]. Low corneal temperature was a risk indicator for glaucoma progression [154], and corneal temperature correlated with BF in the ophthalmic artery [155] and with finger temperature [156]. BF in the ophthalmic artery, in turn, correlated with BF in nailfold capillaries [157]; choroidal BF however (mainly regulated by the autonomic nervous system) was inversely related to finger BF [158].

As a rule, correlations were always present in subjects with VS but not in others. This indicated that in subjects with VS, local regulation was less efficient, and therefore, systemic factors such as perfusion pressure gained more influence.

### The role of gender and body mass index

In the early 1990s, it was known that obesity was a risk factor for many diseases including an IOP increase. However, our patients with NTG tended to be rather slim [159, 160] and were more often female than male [161]. In addition, subjects with low body mass index had lower blood pressure and more often had cold hands [160]. Cold hands or feet occurred at the highest intensity in younger, slimmer women and at lowest intensity in elderly, stouter men [162]. The lower the BP and/or the BMI is, the colder the extremities were [160]. It became clear that low body mass index increases the risk for VS and VS occurs more often in females.

### Silent myocardial ischaemia

We were confronted with a functional vascular problem, measurable in the fingers but obviously also affecting the eye. We therefore asked whether this vascular phenomenon may also affect other organs such as the heart. Episodic asymptomatic ‘silent’ myocardial ischaemia was revealed by 24-h ECGs in many patients with glaucoma, especially normal-tension glaucoma. These episodes did not occur during exercise but at rest, particularly at night, indicating that they were not due to arteriosclerosis but rather due to a functional vasoconstriction [163–165].

### The autonomic nervous system

Our observations raised the question of whether this vascular dysfunction could be a consequence of an autonomic nervous system dysfunction [166]. Whilst the analysis of the beat-to-beat heart rate variation proved an involvement of the autonomic nervous system [167], the dynamic vessel analysis demonstrated a vascular dysregulation also in the non-innervated retinal vessels [168, 169]. This indicated that although VS affected the autonomic nervous system, the imbalance of the autonomic nervous system could not be the sole or even the main cause for the vascular dysfunction.

### The vascular endothelium cells

Beside autonomic innervation and circulating vasoactive hormones, the vascular endothelial cells play a crucial role in the local regulation of BF. The human body has about 10^13^ capillaries and 10^19^ endothelial cells with a total volume that corresponds to the liver. In the past, the vascular endothelium was believed to be just a simple semi-permeable membrane lining the inner part of arteries, veins and lymphatic vessels. Today, we know that endothelial cells interact with immune cells and regulate haemostasis, angiogenesis and, in particular, the vascular tone [170]. They play also a dominant role in the regulation of OBF [171]. In humans, endothelial dysfunction is one of the first clinically detectable alterations in the development of atherosclerosis. Interestingly, however, we found an endothelial dysfunction with the retinal vessel analyser [172] (Fig. 3d) also in non-arteriosclerotic subjects with VS [2, 165, 168, 173, 174], including some weakness of the blood-retinal barrier [175]. The cause of this endothelial dysfunction in these healthy and often very sportive subjects with VS was unclear.

## Altered expression patterns in blood cells

At that time, we had no idea about possible molecular mechanisms that governed VS. Under clinical conditions, the cells of interest, e.g. from the eye, were not amenable. Since we had hypothesised some systemic effects at the level of the gene regulation, we analysed gene expression profiles in peripheral leucocytes applying a ‘gene hunting’ approach and, indeed, found differences in glaucoma patients with VS against controls [176]. The expression profiles were characteristic for adherent leukocytes [177]. Several key pathways have been found to be differently regulated in glaucoma patients with VS including altered stress response, multi-drug resistance and energy metabolism; shifted regulation of transcription, apoptosis and adhesion; deficits in DNA-repair efficacy; blood-brain barrier breakdown; extensive tissue remodelling, etc. [178]. Therein, the enhanced 20S proteasome alpha-subunit levels pointed towards increased oxidative stress [179]. Upregulated MMP-9 and MT1-MMP gene expressions were compatible with the observed partial blood-retinal barrier breakdown [175, 180]. Subjects with VS exhibited differential expression of ABC-transport proteins [181], potentially explaining the altered drug sensitivity [1], and an enhanced expression of ABC 1 transporter was considered to be potentially involved in the vascular dysregulation [182]. The protein expression of AP-2β was increased in both NTG and primary open-angle glaucoma, indicating that this may be related to glaucoma but not to VS [183]. The gene expression profiles were different amongst healthy subjects, patients with high-tension, PEX and normal-tension glaucoma [184]. Interestingly, the expression profiles of NTG patients were very similar to those of healthy subjects with VS [185, 186]. This reinforced the idea of a close relationship between VS and NTG. No differences were found in the genotype frequencies of polymorphisms of the nitric oxide pathway between Caucasian normal and high-tension glaucoma patients [187].

## From vasospasm to vascular dysregulation

Vasospasms have been known in medicine for decades [188] and have also been linked to eye diseases [55, 189], particularly to glaucoma [59, 190]. But the clinical condition we were dealing with in our NTG patients could not be explained by isolated spasms as we knew them, for instance in the retinas of patients with retinal migraine [191]. We were faced with a more global phenomenon, which at that time was described by the term VS [65, 71, 192]. However, this term still suggested mainly abnormal constrictions of arteries. We observed a more general dysregulation, involving arteries, capillaries and veins, including a disturbed autoregulation of OBF [173, 193], abnormal response to hand-grip stimulation [194] or blood gas perturbations [195] and altered responses of both visual fields [196, 197] and ET-1 plasma levels [198] to a change of body position from supine to upright [199]. We therefore considered the term *vascular dysregulation* more appropriate, particularly in the context of glaucoma [9, 190, 200–203].

It also became clear that we needed to separate primary from secondary dysregulation [9, 71, 190, 200, 201, 203]. It turned out that a number of diseases induce secondary vascular dysregulation which can be more local, e.g. due to arteriosclerotic plaques, and more systemic, e.g. due to a high level of ET in the circulating blood.

### Secondary vascular dysregulation

**Fig. 5:**
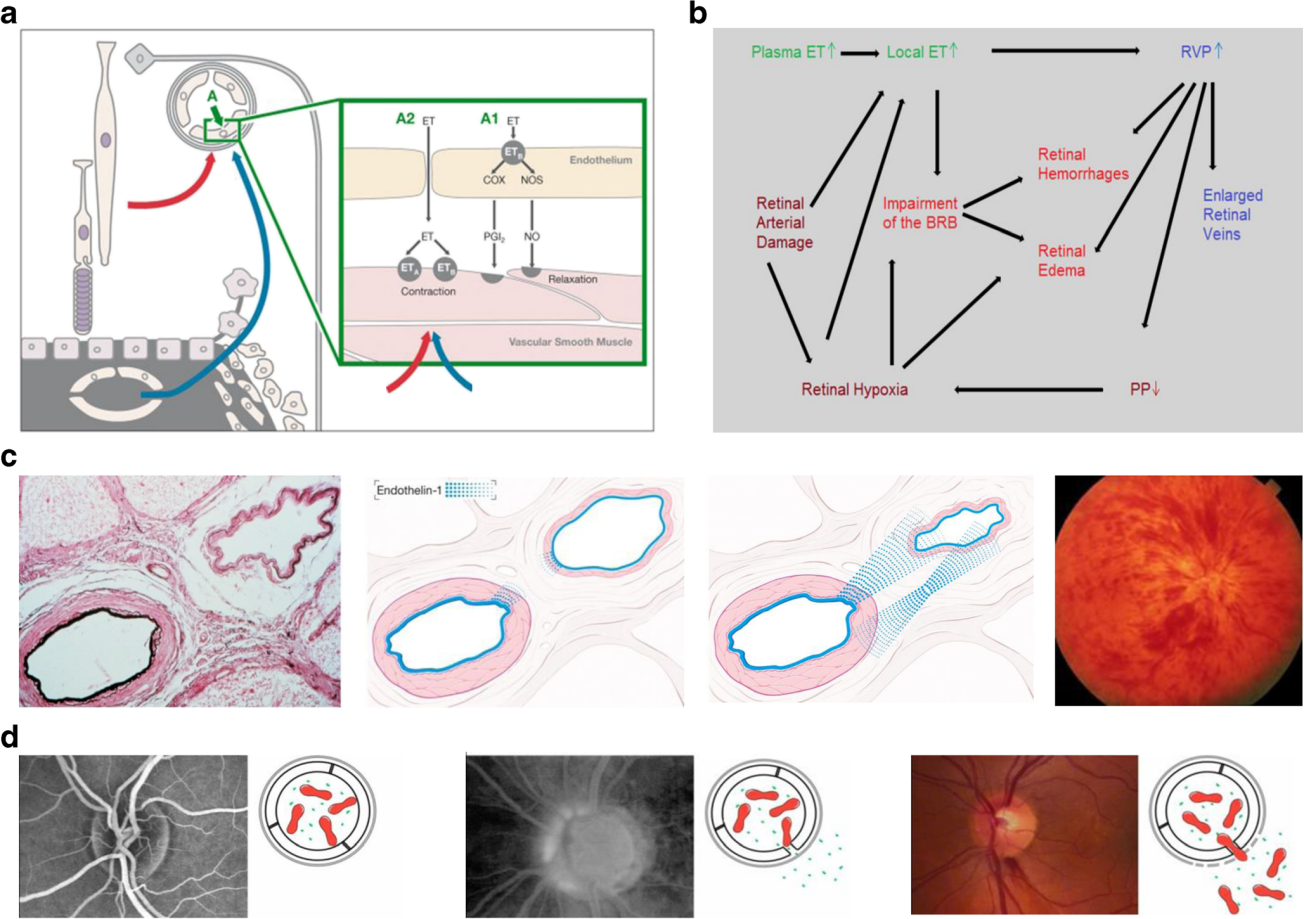
The blood-retina barrier prevents a direct access of circulating molecules (such as a vasoconstrictor endothelin (ET)) to the smooth muscle cells of the retinal vessels. These molecules can however diffuse form the choroid into the optic nerve head (ONH), bypassing this barrier (*blue arrow* in **a**) (modified after ref. [204]). ET can also be produced by the neighbouring hypoxic tissue (*red arrow* in **a**) or diffuse from diseased arteries to adjacent veins (**c**) (modified after ref. [259]). These can potentially increase retinal venous pressure and finally contribute to the pathogenesis of retinal vein occlusion (**b**) (modified after ref. [221]) but also to the pathogenesis of haemorrhages (**d**) (modified after ref. [175])

Secondary vascular dysregulations are neither essential risk factors for normal-tension glaucoma nor part of the FS. But for the sake of completeness, let us briefly discuss them, in particular the role of endothelin (ET). Under physiological conditions ET, a potent vasoconstrictor produced by vascular endothelial cells is mainly released abluminally to regulate local vascular tone [204]. A smaller amount is released intraluminally, contributing to an ET level in the circulating blood [205]. Under pathological conditions (such as inflammations and hypoxia), other cells also produce ET and thereby increase ET levels in the blood. As long as the blood-brain and blood-retina barriers are intact, this has little influence on retinal or brain circulation [206]. However, ET reduces the circulation in the choroid due to fenestrated capillaries [207] and in the optic nerve head due to diffusion from the choroid into the ONH [2, 165, 175] (Fig. 5a). We found increased ET levels in giant cell arteritis [208], rheumatoid arthritis [209], fibromyalgia syndrome [210], multiple sclerosis [211, 212], optic neuritis [213], retinal vein occlusions [214], retinitis pigmentosa [215], Susac syndrome [216] and in patients with cystic macula oedema that responded poorly to anti-VEGF therapy [217]. The border between primary and secondary vascular dysregulation is sometimes vague, which we will discuss by way of the example of multiple sclerosis (MS) further below.

### The primary vascular dysregulation

Vascular dysregulation refers to the regulation of blood flow that is not adapted to the needs of the respective tissue. We determined a vascular dysregulation to be ‘primary’ if no causing disease but a certain genetic predisposition was present [9, 190, 201]. For a detailed description of primary vascular dysregulation (PVD), we refer the reader to a recent comprehensive review [2]. Virtually, all organs, particularly the eye, can be involved. Retinal vessels were stiffer both in patients with glaucoma [218] and in those with PVD [219] and were more irregular [220] in PVD subjects. The vascular response to flicker light was reduced both in patients with glaucoma [174] and with PVD [168, 169] (Fig. 3d). The autoregulation capacity in PVD subjects [173] was reduced, whilst retinal venous pressure was often increased [221]. Subjects with PVD had increased risk for normal-tension glaucoma [59], optic nerve compartment syndrome [222], central serous choroidopathy [223], Susac syndrome [216], retinal artery and vein occlusions [224] and anterior ischaemic neuropathy without atherosclerosis [214]. Further characteristics were their weaker blood-brain and blood-retinal barriers [175, 206] and the higher prevalence of optic disc haemorrhages [206] and activated astrocytes [225].

### Vascular dysregulation in multiple sclerosis

Amongst the subjects with PVD, we relatively frequently saw cases diagnosed by their neurologists as suspected MS, confirmed MS and sometimes Susac syndrome. A certain but not yet well-understood interrelationship was obvious. On the one hand, PVD could imitate some symptoms and signs of MS-like paraesthaesia, temporal pallor of the ONH, visual field defects and prolonged latency in VEP or pathological MRI. In contrast to MS, however, we did not see oligoclonal bands in the cerebrospinal fluid of PVD subjects. On the other hand, MS (like other autoimmune diseases) could lead to a secondary vascular dysregulation [71] with increased ET plasma levels [211] and reduced OBF [212]. MS patients without a history of retrobulbar neuritis had subclinical visual field defects [226], narrower retinal arterioles and wider retinal venules [227], increased rigidity of these retinal vessels [228] and thinning of the macula [229]. During optic neuritis, we observed a transient raise of the ET plasma levels, a reduction of ocular blood flow [213], an improvement of visual function after intake of red wine [230] and a distension of the optic nerve sheaths [231]. A controlled study confirmed the presence of FS symptoms in MS patients [232]. Taken together, we postulated that on the one hand, PVD may not only imitate some MS symptoms but also increase the risk for MS, probably by subclinical cerebral microinfarctions promoting autoimmunity. On the other hand, MS-induced inflammations may evoke secondary vascular dysregulations contributing to chronic progression in a later stage [2].

## The patients are our teachers: clinical symptoms of PVD

Given the complexity of these different parameters and their interactions, one may wonder how we found different signs and symptoms related to PVD. Patients often reported elements seemingly unrelated to their diseases. If other patients reported similar symptoms, we became suspicious and studied a potential relationship with PVD.

### Reduced feeling of thirst

As many of our patients suffered from low BP, we asked them whether they drank enough. Most patients said yes, but not because they were thirsty, only because they knew they had to drink. A controlled study confirmed that subjects with PVD drank more or less enough but indeed had significantly less desire to drink [233]. We assumed that ET (which was slightly increased in subjects with PVD) suppressed the feeling of thirst via upregulation of PGE2 [2]. Correspondingly, the feeling of thirst was also reduced in other diseases with increased ET plasma levels such as in MS [232], retinitis pigmentosa [215, 234, 235], giant cell arteritis [208], polyarthritis [209] or fibromyalgia [210].

### Increased smell perception

Olfactory dysfunction is one of the first symptoms in neurodegenerative diseases. Therefore, we asked our glaucoma patients about smell perception. Whilst patients with glaucoma and with MS had reduced olfactory function [232, 236], subjects with PVD identified odours significantly better than those without PVD [237].

### Prolonged sleep onset time

Visually handicapped subjects, such as patients with advanced glaucoma, potentially lose their circadian rhythms due to disturbed melatonin cycles. Melatonin mediates the arousal system and is also involved in thermoregulation by fine-tuning vascular tone in selective vascular beds [238]. To fall asleep, we need to warm up our feet to a certain temperature [238]. Our patients with PVD often reported long sleep onset time that could be shortened by wearing socks or taking a warm bath. A controlled study confirmed the prolonged sleep onset time [239] and its relation to cold extremities [240].

### Phase delay

Our patients often reported being ‘evening persons’. Subjects with PVD exhibited a normal phase relationship between skin temperature and sleep-wake rhythms [241] but a phase delay of the endogenous circadian system with respect to their habitual sleep-wake cycle [242].

### Cold feet

**Fig. 6:**
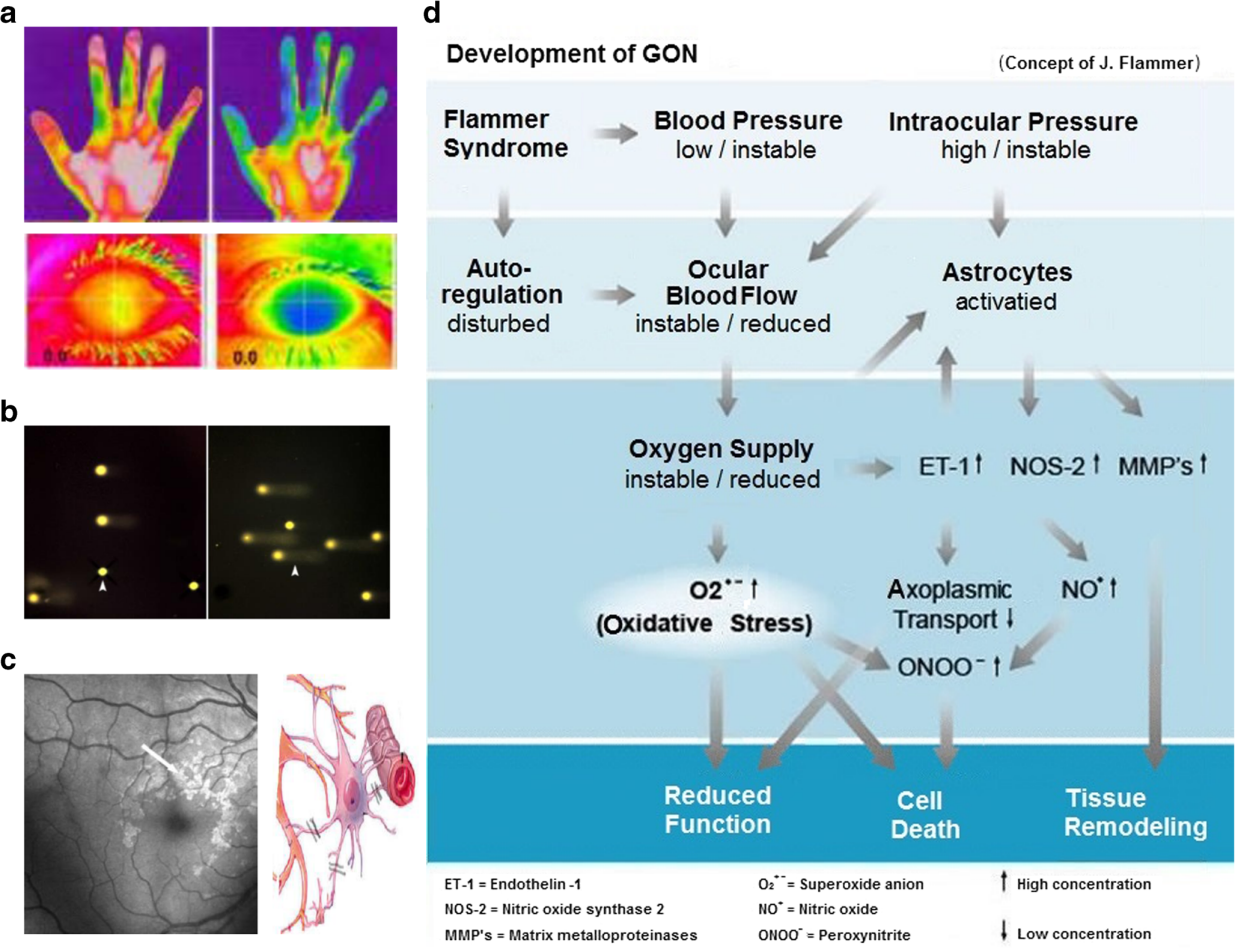
The pathogenetic concept of glaucomatous optic neuropathy (GON). Beside an increased intraocular pressure, a disturbed vascular dysregulation as demonstrated with thermography of the hands and the eyes (**a**) plays an important role (modified after ref. [205]) This leads to an increased oxidative stress as demonstrated with the comet analysis (**b**) (modified after [288]). The activated astrocytes change their gene expression, but also morphology. This increases the backscatter as demonstrated in *red-free photos* (**c**, *left*) (modified after ref. [225]). This also reduces the transport of oxygen from the blood vessels to the neurons (**c**, *right*). The oxygen tension in the axons decreases and in the retinal veins increases. The entire concept is summarised in **d** (modified after ref. [250])

When shaking hands with NTG patients, cold hands were often a striking symptom (Fig. 6a). When asked, these subjects also often indicated having cold feet. When objectively measured, the most constant sign for PVD was an increased temperature difference between lower and higher leg over the entire day but disappearing during sleep. These differences in distal to proximal skin temperatures were independent of the menstrual cycle [243].

### Increased sensitivity

Patients often told us stories that we could sooner or later relate to PVD. One patient felt an earthquake when nobody else around her could feel it. It turned out that she was actually right. Indeed, sensation to vibration was increased [2]. Another patient told us that she became unconscious during a balloon ride. PVD subjects have an increased sensitivity to hypoxia, as it occurs due to low air pressure at high altitudes [244]. One NTG patient experienced a sudden onset of a scotoma during skiing, and three patients became unconscious when jumping into the cold water of the sea. An increased response to cold is typical for PVD. Pain sensation was often reported to be increased [59], and we assumed that an increased level of ET [233, 245] reduced the peripheral pain threshold. Many patients reported increased sensitivity to certain drugs, which could be explained, at least in part, by the differential expression of ATP transport proteins [181]. Sensitivity to psychological stress also seemed high but has not yet been studied scientifically.

### Intensified physical exercise

PVD subjects did physical exercise more often and more vigorous than the average population, and they also seemed to enjoy it more. They particularly often indicated doing running and cycling. Very rarely, however, vasospasms were induced by exercise.

## Ocular manifestations of PVD

Most subjects with PVD were healthy and also free of eye diseases. Nevertheless, they had an altered regulation of OBF [2]. Some PVD subjects developed visible alterations or even diseases, which will now be discussed:

### Activated astrocytes in the retina

In the early 1990s, we observed morphological alterations resembling fine epiretinal gliosis (Fig. 6c). Unlike the real epiretinal gliosis, however, the gliosis-like alterations occurred mainly in the midperiphery of the retina, spared the macula area and never pulled on the retina [246]. Examination with a laser-scanning ophthalmoscope confirmed sharply bordered, patchy retinal alterations in the superficial layers, also called activated retinal astrocytes and Müller cells (ARAM) [225]. Based on occurrence, distribution and phenomenology, we postulated that these patchy alterations were due to an increased backscatter of light by activated astrocytes [247, 248] This phenomenon occurred much more often in subjects with PVD [225], indicating that the blood flow disturbance may have triggered glial cell activation. Examinations with OCT suggested that ARAM may mask retinal nerve fibre loss [249]. Activated astrocytes loose partly their contact with blood vessels and neural axons, preventing normal oxygen transport (Fig. 6c). The consequences are on the one hand a hypoxia in neural cells and on the other hand increased venous oxygen saturation [250].

### Central serous chorioretinopathy

Patients with central serous chorioretinopathy demonstrated a localised delay in arterial filling in the area of the damaged retinal pigment epithelium, frequently associated with dilated capillaries and draining venules. In some patients, localised choroidal ischaemia could be observed in additional areas throughout the central fundus in both diseased eyes and normal fellow eyes [223, 251]. Clinical experience indicated that PVD may be one of the risk factors, probably by causing local hypoxia and oxidative stress. Whilst PVD was more frequent in females, central serous chorioretinopathy occurred more often in young men. A minimal level of testosterone seemed to be a requirement [252].

### Barrier dysfunction and optic disc haemorrhages

The optic nerve head, although part of the central nervous system, lacks classical blood-brain barrier properties. In glaucoma, the blood-brain barrier in and around the optic nerve head was even weaker [175, 253]. We postulated that optic disc splinter haemorrhages were manifestations of a barrier breakdown. If ET opened the barrier on the level of endothelial cells and at the same time MMP-9 weakened the basal membrane, even erythrocytes could escape, leading to the clinical picture of splinter haemorrhages (Fig. 5d). We observed this not only in patients with glaucoma but also in non-glaucomatous subjects with PVD [206].

### Anterior ischaemic optic neuropathy

The ONH is a very vulnerable tissue. It contains non-myelinated nerve fibres consuming more energy than myelinated fibres. In addition, they are exposed to light and to mechanical forces. The ONH lacks a normal blood-brain barrier, providing circulating molecules such as ET or angiotensin direct access to the smooth muscle cells. Whilst the arterial supply stems from the ciliary circulation, the veins empty into the retinal veins, causing the ONH perfusion dependent on retinal venous pressure. The intraocular vessels do not become arteriosclerotic, but the retroocular vessels are heavily involved in arteriosclerosis, probably due to extensive movement by the rotation of the eye. Correspondingly, arteriosclerosis is a strong risk factor for anterior ischaemic optic neuropathy (AION). Nevertheless, AION also occurred in non-arteriosclerotic patients in the presence of PVD. In this case, it occurred nearly always after emotional stress [254, 255]. We observe AION even in children [256] and as a perioperative complication [257, 258].

### Retinal vein occlusion

The classical theory was that retinal vein occlusion (RVO) was a consequence of a thrombus in the retinal vein. In the mid-1990s, we saw seven patients with RVO at the age of <45 years. Extensive evaluations revealed no pathology except increased responses to coldness in the nailfold capillaries and a history of emotional stress [224]. We hypothesised that dysregulation of retinal veins may have played an essential role in the genesis of their RVO, an assumption later supported by the observation of increased ET plasma levels in such patients [214]. Some years later, we reviewed our knowledge of RVO at that time and came to the conclusion that dysregulations of retinal veins must be one of the principal factors in the pathogenesis of RVO [2, 221, 259] (Fig. 5b).

### Cilioretinal arterial occlusion

We observed a cilioretinal arterial occlusion in a 17-year-old young man without any vascular risk factors except FS with an increased retinal venous pressure. With a treatment of a very low dose of nifedipine, the patient remained without relapse [260].

## Choroidal infarction

Occlusions of small end arteries in the choroid with corresponding point-shaped infarctions of the pigment epithelium can frequently be observed in PVD subjects. Extended choroidal infarctions occur less frequent but have also been described [261].

### Retinitis pigmentosa

Retinitis pigmentosa (RP) refers to a group of degenerative eye diseases with a genetic background. Here, OBF seems to influence manifestation and progress. We observed reduced ocular pulse amplitude in RP patients [262]. A review of the literature revealed evidence of OBF reduction in all stages of RP [235], but it remained open whether this was secondary to the retinal degeneration. The retinal-vessel oxygen saturation correlated with structural alterations [263]. Increased ET plasma level [215] pointed towards a primary vascular component. This assumption was then strongly supported by the observation of a significant association of RP with FS [234].

### Optic nerve compartment syndrome

In optic nerve compartment syndrome (ONCS), there is proven segregation of CSF between the intracranial subarachnoid space and the subarachnoid space surrounding the optic nerve. This leads to differences of fluid composition, reduced CSF exchange and an extension of the optic nerve sheath diameter due to increased pressure [222, 264–274]. We observed that ONCS was often related to PVD [2] and that treatment of FS with a low dose of CCBs also reduced ONCS [275].

### Leber's hereditary optic neuropathy

Leber's hereditary optic neuropathy (LHON) is a mitochondrial inherited retinal degeneration affecting predominantly young adult males. It leads to a subacute drop in vision with central visual field defects, starting in one eye and involving the other eye some weeks later. Most of the patients we saw also suffered from PVD [2]. LOHN and PVD may act synergistically. PVD increases the oxidative stress and therefore affects the mitochondrial function, with a particular impact in patients with already weakened mitochondria due to mutations of the mitochondrial DNA [276].

### Decreased vessel density

Vascular regulation is very complex and includes also alternating transient closing of microvessels. An increased ratio of temporally non-perfused vessels can also be a manifestation of a vascular dysregulation giving the impression of a reduced vessel density when blood flow is imaged, e.g. by optical coherence tomography angiography.

## The link between PVD and glaucoma

**Fig. 7:**
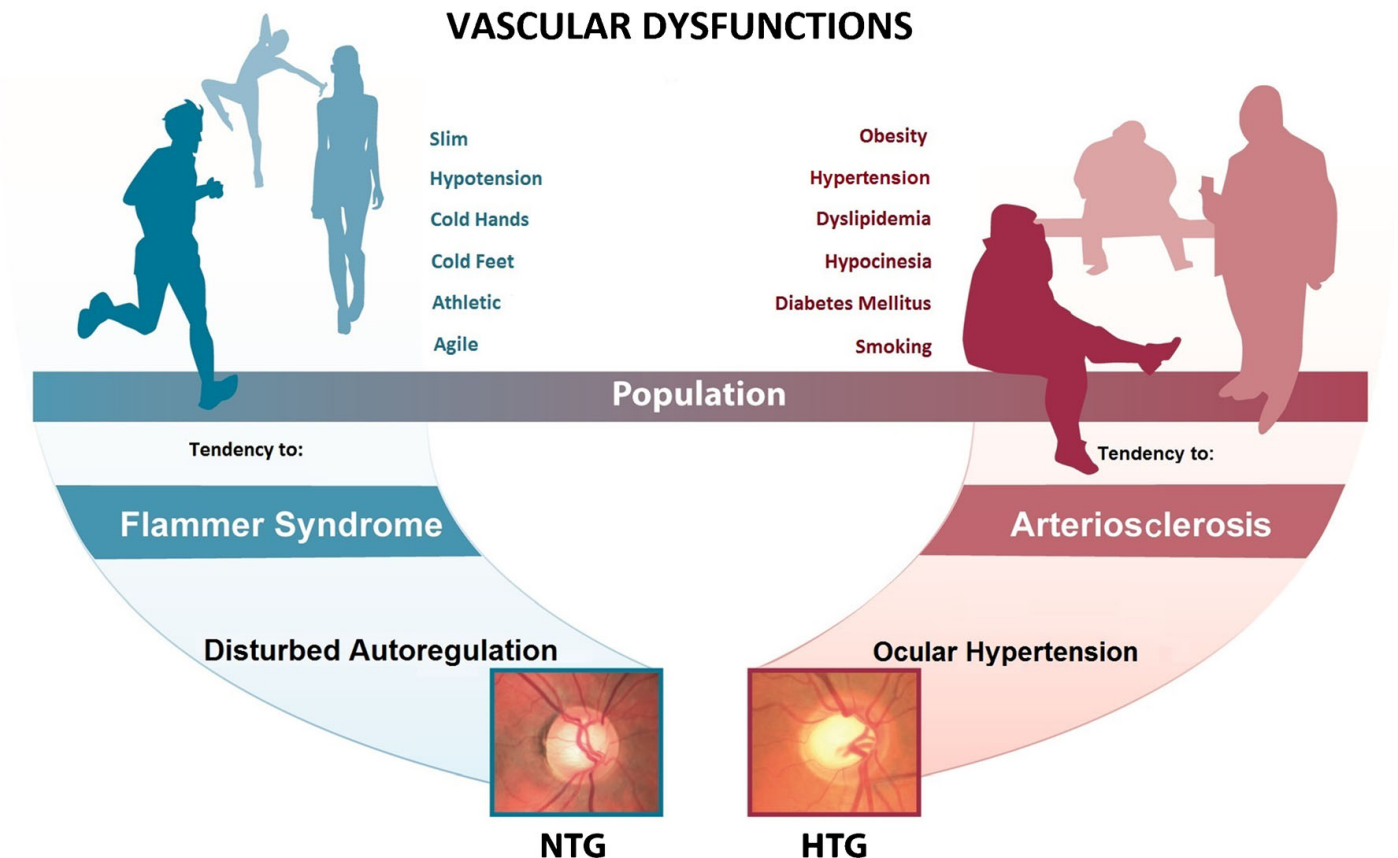
The two extremes in terms of body weight, blood pressure, etc., lead to different types of vascular dysfunctions. Interestingly, both types of dysfunction can lead to very similar end points as exemplified on the development of glaucomatous damage. The Flammer syndrome increases the risk for normal-tension glaucoma (NTG) and the arteriosclerosis (and its risk factors) for high-tension glaucoma (HTG) (modified after ref. [323])

Studies of potential relationships between different health conditions and glaucoma led to much controversy in the literature. One of the main causes of this hot debate was the fact that authors used the term *glaucoma* sometimes for an IOP increase (a risk factor for GON) and sometimes for GON. All the risk factors for arteriosclerosis were also risk factors for an IOP increase (Fig. 7). However, at a given IOP level, they did not further increase the risk for GON. We knew that OBF was altered in glaucoma patients, particularly in those progressing despite normal or normalised IOP [84, 277, 278], but hypoxia, e.g. in the context of a non-arteritic anterior ischaemic optic neuropathy, led to ONH atrophy but not to GON [279] (in contrast to arteritic ischaemic optic neuropathy [280]). We were therefore looking for a potential link [200] between OBF [281] and GON. As oxidative stress [282–284] was a potential candidate [248, 270], we quantified oxidative stress indirectly by measuring DNA breaks with a comet assay [285–288]. Patients with PVD had a significantly higher rate of DNA breaks [285, 289] (Fig. 6b).

We formulated the hypothesis that GON might be the result of repeated small reperfusion injuries [290, 291], in which an unstable oxygen supply causes oxidative stress in the mitochondria of the neural tissue [292]. Oxygen supply is instable if oxygen saturation is unstable, for example, in subjects with sleep apnoea. But oxygen supply was also unstable if the blood supply was unstable [293]. This was the case if IOP fluctuated on a high level or BP on a low level, which may exceed the capacity of autoregulation from time to time [247]. The blood supply was particularly unstable in cases with disturbed autoregulation [173], and this was the case in subjects with PVD [89, 203]. Regulation of blood flow is necessary to adapt to different conditions. Regulation of OBF compensates for varying perfusion pressures (autoregulation), adapts to the retinal activity (neurovascular coupling) and keeps the back of the eye at a constant temperature (thermoregulation) [173, 294]. The regulation was disturbed in some glaucoma patients [295] and in healthy PVD subjects as measured in retroocular vessels [193], in the central retinal artery [89] and in the choroid [194, 203]. This led to the description of a new pathogenetic concept of GON [200, 201, 247, 296, 297] (Fig. 6d) and to new therapeutic approaches [80, 129, 293, 298–302]. It also influenced our preference for certain IOP-lowering drugs [303, 304].

The published effects of drugs on the visual fields of glaucoma patients also generated some confusion. We needed to separate clearly the effect of lowering IOP from direct effects of the drugs on the visual fields. We further needed to separate drug-induced improvements or deteriorations from their impacts on long-term progression of visual fields. As mentioned before, acetazolamide [56, 57], nifedipine [63, 64] and, to some extent, magnesium [301] improved visual fields independently of their IOP-lowering effects. Pindolol [305], carteolol [134, 306] and betaxolol [307, 308] however had a better effect than timolol on the long-term progression, despite a similar or even weaker IOP-reducing effect [303]. It therefore became clear that IOP could not be the only modifiable risk factor for glaucoma [80, 298, 309].

## Psychological characteristics of PVD subjects

The psyche has more influence on the eye than generally assumed [310]. NTG patients showed generally more complaints and were more emotionally unstable than the controls [311, 312]. Therefore, we also searched for psychological characteristics of healthy PVD subjects. A population survey revealed a disposition for socialisation with high-power anger suppression for PVD subjects and for the non-PVD subjects, a tendency towards outwardly expressed anger [313]. Nevertheless, our knowledge about the psychological characteristics of subjects with FS is still very limited.

## Retinal venous pressure

In the 1990s, we already suspected the veins to also be essentially involved in the vascular dysregulations of glaucoma patients [201], but we could not prove this until much later, when we measured retinal venous pressure (RVP) (Fig. 3e,f). Whilst RVP is equal to or slightly above IOP in healthy people, it is often markedly increased in patients with eye or systemic diseases. We postulated that besides mechanical obstructions, the main cause of RVP elevation may be local dysregulations of retinal veins, particularly constrictions induced by endothelin-1 (ET-1) [221]. A local increase of ET-1 can result from a high plasma level, as ET-1 can diffuse from the fenestrated capillaries of the choroid into the ONH, bypassing the blood-retinal barrier [204] (Fig. 5a). A local increase can also result from increased local production, either by a sick neighbouring artery (Fig. 5c) or by hypoxic retinal tissue. Generally, the main factors increasing ET-1 are inflammations and hypoxia, either locally or in remote organs.

RVP was increased in glaucoma [314], and correspondingly, the pulse amplitude in these veins was lower [315]. Eyes with optic disc haemorrhages did not have higher RVP but did have lower retinal arterial pressures in comparison to both the contralateral eyes and eyes of control patients matched for BP [253]. This indicated that the haemorrhages were due to a hypoxia-induced breakdown of the barrier rather than to mechanical ruptures of a retinal vessel [206]. RVP was increased in diabetic retinopathy [316] and in high-mountain disease [244]. RVP was, as expected, increased in eyes with RVO. Surprisingly, RVP was also increased in the clinically non-affected fellow eyes [317]. This contradicted the theory of a thrombus as the primary cause of RVO and supported the hypothesis of a local vasoconstriction of retinal veins at their exit (Fig. 5b,c). RVP was not higher in chronic smokers than in non-smokers [318] but was increased in subjects with FS [319], and CCBs reduced RVP in these FS-positive subjects [320]. Based on these observations, we considered an increased RVP less a consequence of structural alterations but more of a dysregulation of retinal veins. FS seemed to be one of the causes for such a venous dysregulation. A high RVP decreases perfusion pressure, which heightens the risk for hypoxia. A high level of RVP may not only be a consequence but also a potential cause of a RVO. This is because high RVP increases hypoxia and hypoxia stimulates ET production and thereby increases RVP, causing a vicious circle. An increase of RVP also elevates transmural pressure, which in turn heightens the risk for retinal oedema. Patients with RVO-related macular oedema that did not respond to anti-VEGF therapy showed an increase of plasma ET after treatment [217], indicating that hypoxia was a main player in such cases. Narrow retinal arteries and, particularly, dilated retinal veins are known risk indicators for future cardiovascular events [165]. Because the major cause for such a retinal venous dilatation is an increased RVP, RVP may likely turn out to be an even stronger predictor [221].

## Flammer syndrome

The term PVD was better than VS but still not satisfactory, as it became more and more obvious that a syndrome existed that encompassed a holistic altered response of the body including vascular aspects and other signs and symptoms. In addition, PVD was often confused with posterior vitreous detachment. For this reason, one of us (KK), together with some other authors, introduced in 2013 the new term Flammer syndrome. After the term had already been mentioned in different contexts [297, 321], it was elaborated in details in reviews [1, 3] and in book chapters [322].

**Fig. 8:**
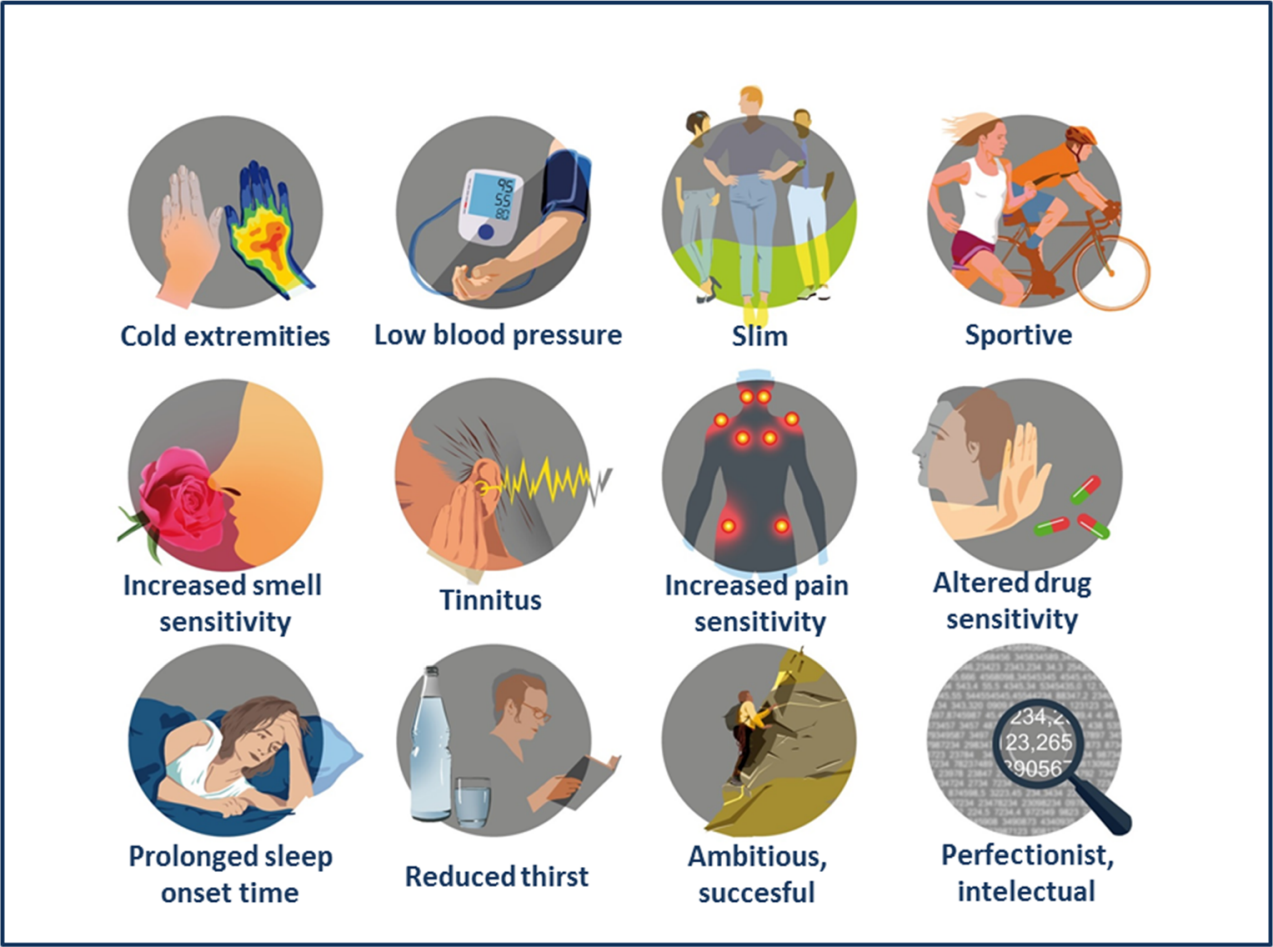
Illustrations of frequent, but not compulsory, symptoms of subjects with Flammer syndrome (modified after ref. [3])

FS describes a phenotype characterised by the presence of PVD [2] together with a cluster of symptoms and signs that may occur in healthy people as well as people with disease. Typically, the blood vessels of the subjects with FS react differently to a number of stimuli, such as cold, physical, chemical or emotional stress. Nearly all organs, particularly the eye, can be involved. Although the syndrome potentially has some advantages for the person affected, e.g. less likelihood becoming arteriosclerotic, FS potentially contributes to certain diseases, such as normal-tension glaucoma. The syndrome occurs more often in women than in men, in slender people more often than in obese subjects, in people with indoor rather than outdoor jobs and in academics more often than in blue-collar workers [279]. Affected subjects tend to have cold extremities, low blood pressure, prolonged sleep onset time, shifted circadian rhythm, reduced feeling of thirst, altered drug sensitivity and increased general sensitivity, including pain sensitivity (Fig. 8). The plasma level of endothelin-1 is slightly increased, and the gene expression in lymphocytes is quantitatively changed. In the eye, the retinal vessels are stiffer and their spatial variability is larger; the autoregulation of ocular blood flow is decreased. Glaucoma patients with FS have an increased frequency of optic disc haemorrhages, activated retinal astrocytes, elevated retinal venous pressure, optic nerve compartmentalisation, fluctuating diffuse visual field defects and elevated oxidative stress.

## Conclusion

The evolution of the present knowledge of FS stretched over several decades. It started with the observation of unexplained phenomena in the outcome of perimetry in glaucoma patients pointing towards the role of OBF. As OBF measurements were unsatisfactory at that time, nailfold capillaroscopy turned out to be a useful substitute until new methods to quantify OBF were introduced. The dynamic retinal vessel analyser finally allowed demonstrating vascular dysregulation. In vitro examinations of ocular vessels and analyses of gene expression of lymphocytes rendered an important basis for understanding the vascular dysfunction. Careful analysis of related symptoms and signs stimulated the initiation of a number of scientific studies. With growing knowledge, the terminology changed from vasospasm over vasospastic syndrome to primary vascular dysregulation and finally to Flammer syndrome.

Additional research proved the involvement of FS not just in glaucoma but also in many other eye diseases such as vascular occlusions and retinitis pigmentosa, but also in systemic diseases such as MS. Establishing the risk for related diseases may lead to predictive and preventive diagnostics, and treatment tailored to the person. This may be particularly relevant for young FS individuals.

The FS is obviously not a new phenomenon. We can only speculate why it has not been recognised earlier. Several aspects may have been jointly responsible: (a) we focus often just on one organ; (b) we tend to prefer monocausal aetiologies; (c) BF dysfunction due to structural changes are easier to observe then changeable functional dysregulations; (d) we tend to extrapolate relationships, although they are often *U*-shaped; and (e) methods to measure BF in vivo or to test blood vessels ex vivo and to quantify gene expression have been markedly improved in the last few decades.

We emphasise that what we described here is only the state of present knowledge. We are convinced that the future will add additional aspects and also correct some of the present assumptions. Science is an ongoing process, and therefore, the understanding of the syndrome and its impact in medicine will further develop. However, our patients suffering from FS symptoms and FS-related diseases deserve to benefit now from the present knowledge, even though it is still limited. Patients are very pleased when they realise that they do not suffer from numerous independent symptoms and sings but rather from one syndrome. They are thankful for information on how they can improve the situation by adapting lifestyle and nutrition. A communication between the different physicians involved makes it possible to avoid unfavourable drugs and, if necessary, to replace them by a treatment adapted to FS in terms of both drug selection and dose.

## Abbreviations:

ABC, ATP-binding cassette; ARAMs, activated retinal astrocytes and Müller cells; BF, blood flow; BMI, body mass index; BP, systemic blood pressure; CCBs, calcium channel blockers; CDI, colour Doppler imaging; DNA, deoxyribonucleic acid; ECG, electrocardiogram; ET, endothelin; FS, Flammer syndrome; GON, glaucomatous optic neuropathy; HTG, high-tension glaucoma; IOP, intraocular pressure; LHON, Leberʼs hereditary optic neuropathy; MMP, metalloproteinase; MRI, magnetic resonance imaging; MS, multiple sclerosis; NTG, normal-tension glaucoma; OBF, ocular blood flow; OCT, optical coherence tomography; ONCS, optic nerve compartment syndrome; ONH, optic nerve head; PVD, primary vascular dysregulation; RVO, retinal vein occlusion; RVP, retinal venous pressure; VEGF, vascular endothelial growth factor; VS, vasospastic syndrome
